# The continued problem of URL decay: an updated analysis of health care management journal citations

**DOI:** 10.5195/jmla.2022.1456

**Published:** 2022-10-01

**Authors:** Susan Howell, Amber Burtis

**Affiliations:** 1 showell@siu.edu, Cataloging and Metadata Librarian, Southern Illinois University Carbondale, Library Affairs, Carbondale, IL.; 2 amber.burtis@siu.edu, Health Sciences Librarian, Southern Illinois University Carbondale, Library Affairs, Carbondale, IL

**Keywords:** URL decay, URL availability, health care management, citation analysis

## Abstract

**Objective:**

This study updates a 2009 study which examined uniform resource locator (URL) decay in health care management journals and seeks to determine whether continued URL availability relates to publication date, resource type, or top-level domain. The authors also provide an analysis of differences in findings between the two study periods.

**Methods:**

The authors collected the URLs of web-based cited references in articles published in five health care management source journals from 2016 to 2018. The URLs were checked to see if they were still active and then analyzed to determine if continued availability was related to publication date, resource type, or top-level domain. Chi-square analysis was conducted to determine associations between resource type and URL availability, and top-level domain and URL availability. A Pearson's correlation was conducted to determine the relationship between publication date and URL availability.

**Results:**

There were statistically significant differences in URL availability across publication date, resource type, and top-level domain. Domains with the highest percentage of unavailable URLs were .com and .net, and the lowest were .edu and .gov. As expected, the older the citation, the more likely it was unavailable. The overall percentage of unavailable URLs decreased from 49.3% to 36.1% between studies.

**Conclusion:**

URL decay in health care management journals has decreased in the last 13 years. Still, URL decay does continue to be a problem. Authors, publishers, and librarians should continue to promote the use of digital object identifiers, web archiving, and perhaps study and replicate efforts used by health services policy research journals to increase continued URL availability rates.

## INTRODUCTION

This study is an update of a study published in 2009 which examined uniform resource locator (URL) decay in health care management journals [1]. The original study determined the availability of 2,011 web-based cited references in articles published in 5 health care management source journals from 2002 to 2004. This study follows the original study methodology using the same 5 health care management source journals 13 years later (from 2016 to 2018) and seeks to determine whether continued URL availability relates to publication date, resource type, or top-level domain. Using the original findings, the authors were able to analyze differences in findings between the two study periods to determine if there were any statistically significant differences.

The original study found that URL decay was a serious problem in health care management journals. Half of the web resources in that study could not be located at the cited URL, with older articles being more likely to have inactive URLs. Whether a URL was active varied by top-level domain but not resource type (i.e., journal, government document, website, miscellaneous). The domain extensions with the largest percentage of inactive URLs were the .com (53%), .gov (51.6%), and .org (47.5%) extensions. The authors of that study were able to find 59.8% of all inactive URLs using the Wayback Machine of the Internet Archive, 48.8% using Google, and 39% using the websites' search functions [1].

This updated study is important because URL decay continues to be an issue of concern to the scholarly community. Numerous calls to remedy the issue have been made [2-16], including the original study [1], but little is known about the change in URL decay over time in one specific academic discipline. A review of the literature on URL decay finds that most papers on the topic primarily address URL inaccessibility at one snapshot in time [2-7], URL inaccessibility at one snapshot in time for a specific discipline (i.e., computer science, information science) [8-9] or a specific journal (i.e., *American Political Science Review, Journal of the Medical Library Association*) [10-11]. A smaller number of studies have looked at content changes in a sample of URLs [12-16], but no other studies known to the authors of this study have followed a similar methodology to this study by comparing data from a specific discipline over two distinct time periods. For this reason, this study offers a unique perspective on the literature of URL decay.

## METHODS

### Data Collection

The URLs of web-based resources cited in articles published in 5 health care management source journals from 2016 to 2018 were compiled in a Microsoft Access database. The citations were gathered from 5 source journals: *Health Affairs, Health Services Research, Health Care Management Review, Journal of Healthcare Management*, and *Medical Care Research and Review*. These journals cover the health care management field comprehensively, as confirmed by surveys of health care management researchers as well as the authoritative Medical Library Association Public Health/Health Administration Core Public Health Journal Project list [17]. The URLs had been collected by the authors as part of another research study updating a healthcare management mapping study [17]; thus the 13-year gap between studies is an artifact of that study. In total, 51,758 citations were compiled in that study. Information about the resource (i.e., source journal, date, type of resource, URL, URL domain extension, availability) was entered into the database manually. For the purpose of this study 10,319 web-based resource citations were extracted and analyzed. The accessibility of each resource at the listed URL was checked in August 2020 and again in August 2021.

Each web-based resource was manually searched using the cited URL to determine if the link was active or inactive. The URL was recorded as active if it brought the authors to the resource cited in the journal reference or if the URL automatically redirected, thereby connecting the authors with the resource. To match the protocol of the previous study the resource had to match the cited date to be considered active. If the resource was revised or was an updated edition it was considered inactive. It is important to locate the originally cited article because researchers rely on references to find original sources and additional information [17]. Any URL not connecting the authors with the cited resource was considered inactive. If the authors hit a pay wall during the URL search (i.e., authentication was required or use was restricted), it was considered inactive for all URLs except for journal articles. The rationale for not counting journal articles as inactive in these situations was that a patron could request the article through interlibrary loan in cases where their institution did not have an active subscription.

If the URL was inactive, further checking was done to see if the cited resource could be retrieved by various other searching strategies. These strategies were chosen to match the methodology of the previous study. The title as well as analysis of the URL were used in attempts to locate the resource. First the title was entered into the search box of the corresponding homepage of the cited resource. Next, the URL was searched by shaving extensions working from right to left with each backslash. Additionally, the title of the resource was searched using Google as the search engine. Finally, the Wayback Machine of the Internet Archive was used to locate the resource. The Wayback Machine crawls the Internet daily to capture and then archive versions of web pages [18]. When a URL was located using the Wayback Machine each individual web capture was viewed to locate the cited content. If the authors were able to gain access to the resource with the same cited date, it was considered recovered. Even if the resource was found using one recovery method, each method of recovery was tested, and the results recorded.

### Statistical Analyses

Statistical analyses were performed using Excel. The authors analyzed the differences across journal article publication date, resource type, and top-level domain with URL availability. Additionally, data from the original study was compared to the data collected in this study. The level of significance was set at .05 for all statistical analyses done in this study. Throughout this study, URL decay is synonymous with the percentage of inactive URLs and was calculated by dividing the number of inactive URLs by the number of total URLs. The percentage increase or decrease between two percentages was calculated by dividing the difference between the two percentages by the original number.

A Pearson's correlation coefficient was calculated to determine if there was a relationship between the number of URLs and the percentage of URL inactivity. A Pearson's correlation was also used to check if the age of a source journal article's publication date and URL availability were related.

A Chi-square analysis relating resource type to URL availability was performed. Each URL was recorded as one of four resource types (i.e., journal, government, web, miscellaneous). These categories correspond to the categories used in the authors' earlier study [17]. Journal resource types included scholarly and trade journals as well as government published serials. Government resource type was used for anything other than serials that were published by a government agency at the local, regional, national, or international level (e.g., United Nations). If a resource had a URL that was not government-sponsored, it was considered a web resource. The miscellaneous resource type included resources that did not fit in the other categories (i.e., dissertations, software, newspapers, etc.).

A Chi-square analysis was used to examine the relationship between top-level domain and URL availability. The top-level domain for each resource was recorded as .com, .edu, .gov, .net, or .org. If the URL did not use one of those extensions, the researcher used the cited URL to connect to the resource and determined which one of these categories would be the closest match. Most often, the “About Us” information was used. Corporate entities were considered .com, universities and educational institutions were considered .edu, government-sponsored sites were considered .gov, and nonprofit organizations were considered .org.

### Comparison with Original Study

The original 2009 study included citations from articles published from 2002 to 2004 and the URLs were checked twice in 2007, once in March and once in August. Similarly, data from the current study were also from articles published during a three-year period (2016 to 2018) although URLs were checked one year apart in August 2020 and in August 2021. This methodology was chosen to replicate the previous study's final check at 3 years post-publication, and also to observe the change in URL decay within the time span of 1 year. Additionally, URLs could have been temporarily unavailable the first time they were checked, and the authors wanted to account for that possibility.

Paired sample t-tests were performed using Excel to compare the percentage of active and inactive URLs by year of publication and by domain extension between the two studies.

## RESULTS

The data from this study consisted of 10,319 URLs extracted from the cited references of 5 source journals which included 51,758 total citations. The initial check for URL availability found that 27.7% (2,860) of the citations could not be located with the cited URL. When the URLs were checked again one year later, 36.1% (3,726) of the citations could not be found at the cited URL. Within the time span of 1 year, there was a 30.3% (866) increase in the percentage of inactive URLs. Only 48 URLs (0.47%) were inactive the first time they were checked and then found active on the second check 1 year later. This may have been because the servers were temporarily unavailable, or the web sites were down when the initial check was done. The data from the 2021 final check were used in the tables and analysis to follow.

### URL Decay by Journal Title

Table 1 shows the number of active and inactive URLs by journal title. *Health Affairs*, which had the greatest number of total URLs, had the lowest percent of inactive URLs (i.e., URL decay). *Health Care Management Review* had the highest percent of URL decay. *Health Affairs, Health Services Research*, and *Medical Care Research and Review* (9,524 total URLs with 3,315 inactive) are health services policy research oriented, while *Health Care Management Review* and *Journal of Healthcare Management* (795 total URLs with 411 inactive) are practitioner oriented. The health services policy research journals in this study had lower overall URL decay than practitioner-oriented journals (34.8% vs. 51.7%, p = 0.18). No significant difference was found between the total number of URLs and the percentage of inactive URLs by journal (*r*(3)=0.78, *p* = 0.124). For that reason, the number of total URLs for a specific journal does not account for the difference between the percentage of inactive URLs in health services policy research journals and practitioner-oriented journals.

**Table 1:** Number of active and inactive uniform resource locators (URLs) by journal title active uniform resource locators (URLs) by journal title

Journal Title	Active URLs (%)	Inactive URLs (%)	Total URLs
*Health Affairs*	4,707 (71.1)	1,909 (28.9)	6,616
*Health Care Management Review*	72 (38.7)	114 (61.3)	186
*Health Services Research*	1,143 (49.5)	1,167 (50.5)	2,310
*Journal of Healthcare Management*	312 (51.2)	297 (48.8)	609
*Medical Care Research & Review*	359 (60.0)	239 (40.0)	598
Total	6,593 (63.9)	3,726 (36.1)	10,319

#### URL Decay by Year of Journal Article Publication

Table 2 shows URL decay (i.e., percent of inactive URLs) by year of journal article publication. The older the URL, the higher the number of unavailable URLs. There was a statistically significant positive relationship between the age of journal article publication and URL availability (*r*(10,319)=0.054, *p* = .00001). The highest percentage of unavailable URLs were in 2016 and the lowest were in 2018.

**Table 2:** Number and percentage of active and inactive URLs by year of publication

Year of Article Publication	Number of Articles	Active URLs (%)	Inactive URLs (%)	Total URLs
2016	448	2,010 (59.7)	1,359 (40.3)	3,369
2017	429	2,130 (65.8)	1,105 (34.2)	3,235
2018	533	2,453 (66.0)	1,262 (34.0)	3,715

#### URL Decay by Resource Type

Table 3 shows URL decay (i.e., percent of inactive URLs) by type of resource (i.e., government, journal, web, miscellaneous). There was a significant difference between resource type and whether the URL was active (χ^2^=20.13, *df*=3, *p* < .0002, *n*=10,319). The resource type with the highest percentage of unavailable URLs was websites, followed by government resources.

**Table 3:** Number and percentage of active and inactive URLs by resource type

Resource Type	Active URLs (%)	Inactive URLs (%)	Total URLs
Government	3,242 (65.4)	1,715 (34.6)	4,957
Journal	220 (70.3)	93 (29.7)	313
Web	3,117 (62.0)	1,914 (38.0)	5,031
Misc.	14 (77.8)	4 (22.2)	18

#### URL Decay by Top-level Domain

Table 4 shows URL decay (i.e., percent of inactive URLs) by top-level domain. There was a significant difference between domain level and whether the URL was active (χ^2^=80.46, *df*=4, *p* < .00001, *n*=10,319). The domains with the highest percentage of unavailable URLs were .net and .com, and the domains with the lowest were .edu and .gov.

**Table 4:** Number and percentage of active and inactive URLs by top-level domain

Extension	Active URLs	(%) Active	Inactive URLs	(%) Inactive	Total URLs
.com	729	53.7%	628	46.3%	1,357
.edu	284	68.1%	133	31.9%	417
.gov	2,990	66.1%	1,536	33.9%	4,526
.net	32	49.2%	33	50.8%	65
.org	2,558	64.7%	1,396	35.3%	3,954

#### Recovery of Inactive Links

Of the 4 recovery methods used to retrieve the cited resources with inactive URLs, the Wayback Machine was the most effective method, recovering 2,143 (76.2%) of the 2,812 URLs that were inactive in both checks. Searching the title in Google was the second most effective method, recovering 2,044 (72.7%) URLs. The homepage search recovered 828 (29.4%) of the URLs. The truncated or shaved search was the least effective, recovering 285 (10.1%) URLs. There were still 192 (6.8%) that the authors were unable to locate by any of the recovery methods chosen in this study.

#### Comparing the Two Studies

The number of URLs collected and analyzed for this study (10,319) was 5.1 times greater than the original data collected in 2009 (2,011), even though both studies examined a sample of 3 years of URLs from the same 5 journals. In this same time period the number of articles also increased, though to a lesser degree from 989 to 1,410. Table 5 compares the percentage of active and inactive URLs by journal title between the 2 studies. There is a significant difference in the percentage of inactive URLs between the 2 studies (*t*(4)=2.776, *p* = 0.018). Therefore, this study concludes that URL decay decreased significantly over the 13 years between the two studies. *Health Affairs* continued to have the lowest percentage of unavailable URLs, and *Medical Care Research & Review* showed the greatest improvement with a 36.5% decrease in the percentage of inactive URLs.

**Table 5:** Number and Percentage of active and inactive URLs by journal title (2007 vs. 2021)

Journal Title	Active URLs (%) 2021	Inactive URLs (%) 2021	Active URLs (%) 2007	Inactive URLs (%) 2007
*Health Affairs*	4,707 (71.1%)	1,909 (28.9%)	876 (54.9%)	720 (45.1%)
*Health Care Management Review*	72 (38.7%)	114 (61.3%)	14 (35.9%)	25 (64.1%)
*Health Services Research*	1,143 (49.5%)	1,167 (61.3%)	81 (37.2%)	137 (62.8%)
*Journal of Healthcare Management*	312 (51.2%)	297 (48.8%)	28 (26.9%)	76 (73.1%)
*Medical Care Research & Review*	359 (60.0%)	239 (40.0%)	20 (37.0%)	34 (63.0%)
Total	6,593 (63.9%)	3,726 (36.1%)	1,019 (50.7%)	992 (49.3%)

Table 6 compares the percentage of active and inactive URLs by year of publication. When considered in aggregate, there was no significant difference between the 2 studies for year of publication and URL decay (*t*(2)=2.924, *p* = 0.1). However, the trend in the rate of URL decay shows the percentage of inactive URLs decreasing at a slower rate in the current study than in the first study. Between 2016 to 2018, there was a 15.6% decrease in the percentage of URL inactivity (from 40.3% to 34.0%). Comparing 2002 and 2004, there was a 35.8% decrease in the percentage of URL inactivity (from 61.1% to 39.2%).

**Table 6:** Number and Percentage of active and inactive URLs by year of journal publication (2007 vs. 2021)

Year of Publication	Active URLs (%)	Inactive URLs (%)	Total URLs
2016	2,010 (59.7%)	1,359 (40.3%)	3,369
2017	2,130 (65.8%)	1,105 (34.2%)	3,235
2018	2,453 (66.0%)	1,262 (34.0%)	3,715
			
2002	187 (38.9%)	294 (61.1%)	481
2003	241 (43.0%)	319 (57.0%)	560
2004	564 (60.8%)	363 (39.2%)	927

Table 7 compares the percentage of active and inactive URLs by domain extension and by study. When considered in aggregate, there was no significant difference between the 2 studies for domain extension and URL decay (*t*(5)=0.921, *p* = 0.4). However, when looking at the domains individually, .edu is the only domain that remained stable. The domain with the greatest percentage decrease for inactive URLs was .gov (34.3%). There was a 12.6% decrease for the .com domain and a 25.7% decrease for the .org domain. The domain with the greatest percent increase for inactive URLs was .net (31.95%).

**Table 7:** Number and Percentage of active and inactive URLs by top-level domain (2007 vs. 2021)

Extension	Active URLs (%) 2021	Inactive URLs (%) 2021	Active URLs (%) 2007	Inactive URLs (%) 2007
.com	729 (53.7%)	628 (46.3%)	124 (47.0%)	140 (53.0%)
.edu	284 (68.1%)	133 (31.9%)	54 (68.4%)	25 (31.6%)
.gov	2,990 (66.1%)	1,536 (33.9%)	326 (48.4%)	348 (51.6%)
.net	32 (49.2%)	33 (50.8%)	16 (61.5%)	10 (38.5%)
.org	2,558 (64.7%)	1,396 (35.3%)	390 (52.5%)	353 (47.5%)

## DISCUSSION

As expected, the older the URL, the higher the percentage of decay. The total number of URLs in this sample was 5.1 times greater than the original study even though both studies examined a sample of 3 years of the same 5 journals. This large increase in the total number of URLs cited in the scholarly literature has also been reported by numerous other studies, including a recent paper analyzing URL decay in the biomedical literature [19], and papers mapping the literature of healthcare management [17], dental hygiene [20], and pediatric nursing [21].

There were statistically significant differences in URL availability across resource type and top-level domain in this study. Websites were the resource type with the most URL decay. Domains with the most decay were .com and .net and the domains with the least decay were .edu and .gov.

Overall, URL decay decreased from 49.3% to 36.1% between the two study periods. Even though this is a statistically significant improvement from the previous study, this still means that over one-third of all citations to URLs in the latest study became irretrievable over a relatively short period of time.

The trend in URL decay in this study appears to be more stable overall than the original study, with only a 15.6.% decrease in the percentage of inactive URLs from 2016 (40.3%) to 2018 (34.0%) compared to a 35.8% decrease during a similar time span in the original study. Furthermore, even though there was a longer time between rechecking the links in this study (6 months vs. 1 year), there was still a 30.3% increase in the percentage of inactive links between checks.

Both studies showed significant differences in URL decay between top-level domains, but there was no significant difference between the two studies. URL decay for the .edu domain extension remained stable, standing out as the domain with the lowest percentage of decay across both studies. This is not surprising considering the relative stability of educational institutions when compared to other sources. Interestingly, the .gov domain extension saw the largest decrease in the URL decay, perhaps due to web archiving and permanent URL initiatives by government agencies [22]. The .net domain extension saw the largest increase in URL decay between studies and .com extension decay was high across both studies. This pattern is not surprising considering both extensions are commercial sites where information about products and services are updated and changed regularly. Even if a URL was accessible, if the original content has been updated or changed it was considered to suffer from “content drift” and was considered unavailable (i.e., decayed) [19].

To decrease URL decay of citations to web resources, it has already been suggested that publishers, editors, and authors should work together to require authors to retain a digital backup or printed copies of cited web resources, advocate for the inclusion of cited web resources in online archives, and check URLs before publication for typos [11]. Inclusion in an online archive is not a panacea, however, as the Wayback Machine will remove pages at the owner's request. Furthermore, there is not much evidence that online archiving of cited web resources has become common practice in academic publishing. Beyond a 2004 study that looked at the digital information archiving policies of the 100 highest impact medical and scientific periodicals [23], the authors could find no other studies that looked at publisher policies related to cited web resource archiving. Furthermore, the 2004 study found that only 1% of journals provided recommendations on how to archive cited web resources and none required DOIs [23]. No follow-up study to date has been published to determine if this rate has changed, but a similar study examining the policies of health care management journals would be warranted. It should be noted that none of the 5 journals used in this study have author policies or guidelines for archiving cited web resources [24-28].

The findings of this study are relevant to health care managers who need to make evidence-based decisions regularly. Citations are the backbone of a journal article. If citations are irretrievable, practitioners may be unable to apply research findings to their own specific health care setting. The findings are also applicable to health services researchers who are increasingly following open science principles, which promote best practices around reproducibility, transparency, and research data management [29].

The original 2009 study provided an extensive list of possible resources for combating the problem of URL decay, including digital object identifiers (DOIs), uniform resource names (URNs), persistent uniform resource locators (PURLs), robust hyperlinks, institutional repositories, and personal web archiving tools like WebCite and the Wayback Machine of the Internet Archive [1]. The authors of that study argued at the time that WebCite, an on-demand Internet archiving service which is now defunct, was the most promising of the web archiving tools. Of the other solutions offered, increased DOI use for web resources that are eligible for DOI attribution (i.e., journal articles and government publications) may have contributed to the decrease in URL decay between studies.

Of relevance to reference librarians, the Wayback Machine of the Internet Archive was highly effective in recovering inactive URLs in this study. The Wayback Machine archives previous versions of web pages, so it has an edge over the other methods, including Google, in locating the cited version of a resource. Librarians should promote the use of the Wayback Machine for researchers attempting to track down web citations. It also continues to be the most widely used and longstanding web archiving service, and so the authors recommend it as the most sustainable solution to the problem of URL decay. Another available option that has emerged in recent years for web archiving is perma.cc. It was developed by Harvard Library Innovation Lab and allows authors to archive their cited web resources. This is a free service for up to ten URLs per month for individual authors and unlimited access for academic institutions and courts. Other organizations can purchase subscriptions [30]. Perma.cc may prove to be a useful tool, however, it would require the action of the authors to create an account and upload their link(s). There is not likely to be one single solution to the problem of URL decay. It will most likely involve a multipronged effort between librarians and publishers to initiate web archiving and DOI attribution policies and to promote tools that support these efforts.

## CONCLUSION

URL decay in health care management journals remains high at 36.1%, although not as high as the previous study where 49.3% of all URLs were not available 3 to 5 years after the date of citation. This 26.8% overall decrease in URL decay in the 13 years between studies is promising, though it can still be concluded that URL decay in health care management journals is still an issue since it is not negligible. Other studies examining URL decay in various other fields have found a similar pattern of decreasing URL decay over a similar time period, so this study supports that overall trend. It should be noted, however, that these studies did not use the same set of journals as each other, nor did they follow the same methodology of this study. For instance, independent studies looking at URL decay in Library and Information Science journals found 45.4% of URLs to be inaccessible in 2003 [31], 31% in 2007 [32], 27% in 2010 [33], and 23.1% in 2022 [34]. Likewise, 2 different studies of URL decay in Communication journals found 50% of URLs to be inaccessible in 2007 [35], and 15.7% in 2022 [34].

This study is unique because no other study known to the authors has looked at the change in URL decay rate of a specific set of journals over time. Further research should look at changes in publisher policies mandating web archiving or DOIs, especially in the last two decades, to determine if there is any correlation between changes in publisher policies and changes in URL decay. This study did not investigate whether there was a difference in accessibility due to content drift or failure of the link itself. This would also be an area for further research.

## LIMITATIONS

This study analyzed the citation data from five health care management journals over a period of three years. The results should not be generalized to cover all journals over an extended period. The authors' choice of using Google as a search engine may have affected the recovery rate as no one single search engine indexes every resource.

## Data Availability

Data associated with this article are available in the authors' institutional repository at https://opensiuc.lib.siu.edu/morris_data/.

## SUPPLEMENTAL FILES

**NA**
